# Dynamic Prognostic Models for Colorectal Cancer With Liver Metastases

**DOI:** 10.1001/jamanetworkopen.2025.29093

**Published:** 2025-08-27

**Authors:** Qichen Chen, Yiqiao Deng, Kun Wang, Yuan Li, Xinyu Bi, Kan Li, Hong Zhao

**Affiliations:** 1 Department of Colorectal Surgery, State Key Laboratory of Oncology in South China, Guangdong Provincial Clinical Research Center for Cancer, Sun Yat-sen University Cancer Center, Guangzhou, China; 2 Department of Hepatobiliary Surgery, National Cancer Center/National Clinical Research Center for Cancer/Cancer Hospital, Chinese Academy of Medical Sciences and Peking Union Medical College, Beijing, China; 3 Key Laboratory of Carcinogenesis and Translational Research, Hepatopancreatobiliary Surgery Department I, School of Oncology, Beijing Cancer Hospital and Institute, Peking University, Ministry of Education, Beijing, China; 4 Merck & Co Inc, Rahway, New Jersey

## Abstract

**Importance:**

Current prognostic models for colorectal liver metastases (CRLM) primarily incorporate clinicopathologic features assessed at a single time point, resulting in a static risk assessment for individuals. Given that tumor progression is a dynamic process, especially for patients with CRLM, and patients’ data are continuously collected during the follow-up visits, dynamic prediction is a natural model for risk assessments via reflecting the latest prognosis, whenever new marker measurements are available.

**Objective:**

To develop CRLM prognostic models and a clinical web-based tool to facilitate dynamic predictions.

**Design, Setting, and Participants:**

In this retrospective prognostic study, patients with CRLM who underwent resection between January 2014 and January 2021, were included in the training and validation cohorts. Clinicopathologic characteristics and preoperative and postoperative laboratory measurements taken within 12 months after surgery across 9 laboratory markers (carcinoembryonic antigen, carbohydrate antigen 19-9, γ-glutamyl transferase, red blood cell distribution width SD and coefficient of variance, aspartate aminotransferase to platelet ratio index, Fibrous-4 index, S-index, and neutrophil-to-lymphocyte ratio) were collected. Three prediction models for progression-free survival (PFS) and overall survival (OS) based on a functional random survival forest framework were constructed and compared: model A incorporated only clinicopathologic characteristics, model B included clinicopathologic characteristics and preoperative laboratory markers, and model C integrated clinicopathologic characteristics along with longitudinal laboratory markers. Data were analyzed from June 2024 to June 2025.

**Exposure:**

Resection in patients with CRLM.

**Main Outcomes and Measures:**

Performance metrics included area under the receiver operating characteristic curve (AUC) and Brier score (BS).

**Results:**

A total of 976 patients (median [IQR] age, 59 [51-65] years; 612 [62.7%] male) were eligible for this study, with 758 patients in the training cohort (median [IQR] age, 59 [52-66] years; 487 [64.2%] male) and 218 patients in the validation cohort (median [IQR] age, 58 [49-64] years; 125 [57.3%] male).The training cohort included a total of 24 992 longitudinal measurements, and the external validation cohort included 7198 longitudinal measurements. In the external validation cohort, model C demonstrated an improved prognostic capability compared with models A and B, with AUC values of 0.796 (95% CI, 0.740-0.848) for 1-year progression-free survival (PFS), 0.837 (95% CI, 0.768-0.899) for 3-year PFS, and 0.850 (95% CI, 0.780-0.914) for 5-year PFS. The corresponding BSs were 0.246 (95% CI, 0.236-0.261) for 1 year, 0.205 (95% CI, 0.193-0.218) for 3 years, and 0.142 (95% CI, 0.132-0.153) for 5 years. Model C consistently outperformed models A and B for overall survival (OS) prognosis, with AUCs of 0.849 (95% CI, 0.768-0.914) for 1 year, 0.741 (95% CI, 0.667-0.815) for 3 years, and 0.753 (95% CI: 0.656–0.849) for 5 years, alongside BS values of 0.047 (95% CI, 0.045-0.048) for 1 year, 0.178 (95% CI, 0.168-0.195) for 3 years, and 0.144 (95% CI, 0.133-0.165) for 5 years. Additionally, dynamic individualized risk profiles for PFS and OS were developed for patients. A web-based tool was created to facilitate the practical application of these dynamic prediction models for new patients in clinical environments.

**Conclusions and Relevance:**

In this retrospective prognostic study, the dynamic models, along with the web-based tool for personalized prediction, demonstrated improved performance by incorporating multiple longitudinal makers.

## Key Points

**Question:**

Can longitudinal laboratory markers improve prediction models for progression-free and overall survival in patients with colorectal liver metastases (CRLM) after resection?

**Findings:**

In this prognostic study of 976 patients with CRLM, the dynamic prediction models incorporating multiple longitudinal markers consistently outperformed models based on static clinicopathologic and baseline laboratory data in predicting progression-free and overall survival, with improved areas under the receiver operating characteristic curve and Brier scores across multiple time points.

**Meaning:**

These findings suggest that dynamic prediction models offer a practical approach to more precise and personalized risk assessment for patients with CRLM, potentially informing tailored clinical management.

## Teaser

This prognostic study develops and evaluates dynamic prognostic models for progression-free and overall survival using longitudinal data from patients with colorectal liver metastasis

## Introduction

The liver is the most common site of metastasis and the primary cause of death in colorectal cancer (CRC).^1^ The incidence of synchronous colorectal liver metastases (CRLM) is approximately 20% to 30%, with biological behavior significantly worse compared with metachronous liver metastases.^2,3^ Treatment of synchronous CRLM has become a critical factor in improving CRC prognosis. Simultaneous resection of CRC and liver metastases is an effective treatment for appropriate patients with synchronous CRLM, offering better survival odds than staged resection, along with advantages such as lower treatment costs, reduced discomfort from secondary surgeries, and shorter adjuvant therapy windows.^4,5^ However, recognizing that not all patients with CRLM derive benefits from simultaneous resection is pivotal, given that a subset of patients may face suboptimal postoperative survival outcomes, with potential rapid disease progression or mortality.^6^ Early identification and individualized outcome predictions for individuals with such high risk, accompanied by the implementation of requisite interventions and the establishment of comprehensive follow-up protocols, is imperative in clinical management strategies.

In patients with CRLM, many prognostic models have been established.^6,7,8,9^ However, these models are typically static and provide a single risk estimate based on information available at 1 time point. Given that tumor prognosis is a dynamic process, especially for patients with CRLM, and patient data are continuously collected during follow-up visits, dynamic prediction is a natural approach to risk assessments via reflecting the most current prognosis and capturing temporal changes whenever new marker measurements are available. Laboratory markers, which are routinely monitored during treatment and follow-ups, offer potential for dynamic prediction. In patients with CRLM, some commonly used preoperative laboratory markers (eg, carcinoembryonic antigen [CEA],^10^ carbohydrate antigen [CA] 19-9,^11^ γ-glutamyl transferase [GGT],^12^ red blood cell distribution width [RDW] standard deviation [SD],^13,14^ RDW coefficient of variation [CV],^13,14^) and composite laboratory markers (eg, aspartate aminotransferase [AST] to platelet ratio index [APRI],^15^ Fibrous-4 index [FIB-4],^16^ S-index,^17^ neutrophil-to-lymphocyte ratio [NLR]^18^) have been reported to provide good prognostic capabilities. Their accessibility, dynamic monitoring, and associations with prognosis make them promising candidates for dynamic prediction models.

To refine the prognostic process for patients with CRLM undergoing simultaneous resection, we adopted a novel method to jointly model the longitudinal observations of multiple laboratory markers and time to event of interest (eg, progression-free survival [PFS] and overall survival [OS]) using a multivariate functional principal component approach with random survival forest (RSF). This approach has the advantage of handling the associations among longitudinal markers; missed, irregularly observed data; measurement errors; and no prespecified form of the longitudinal trajectory.^19,20,21^ Furthermore, we developed a clinically applicable dynamic prognostic tool based on this model,^22^ designed to dynamically update the patient’s prognosis with new information, thereby offering a valuable resource for ongoing patient management.

## Methods

This prognostic study was reviewed and approved by the ethics committees of the Cancer Hospital, Chinese Academy of Medical Sciences and Sun Yat-sen University Cancer Center. As a retrospective study that did not involve any treatment interventions, it was granted exemption from the requirement for informed consent by the committees. This study adhered to the Transparent Reporting of a Multivariable Prediction Model for Individual Prognosis or Diagnosis (TRIPOD) reporting guideline.

### Patients and Variables

Consecutive patients with CRLM undergoing simultaneous resection between January 2014 and January 2021 were retrospectively identified from 3 independent cancer hospitals. The study variables included 18 clinicopathologic characteristics (eg, age, sex, body mass index, comorbidity, American Society of Anesthesiology score, primary tumor site, tumor differentiation, lymph node metastasis, liver metastasis size, extrahepatic metastases, major liver resection, R0 resection, liver metastasis number, bilobar liver distributions, preoperative chemotherapy, *KRAS* mutation, and adjuvant chemotherapy, AST, alanine aminotransferase [ALT], albumin [ALB]) and 9 specific laboratory markers (tumor markers: CEA and CA19-9; nontumor markers: GGT, RDW-SD, RDW-CV, APRI, FIB-4, S-index, and NLR) reported to provide good prognostic capabilities.^10,11,12,13,14,15,16,17,18^ APRI was calculated as 100 × ([AST / upper normal limit] / platelet count [in ×10^9^/L]).^15^ FIB-4 was calculated as age × AST / (platelet count [in ×10^9^/L] × ALT^1/2^).^16^ S-index was calculated as 1000 × GGT / (platelet count × ALB^2^).^17^ These 9 specific laboratory markers relied on the collection of 11 hematological markers (CEA, CA19-9, RDW-SD, RDW-CV, neutrophil, lymphocyte, platelet count, GGT, AST, ALT, and ALB). The 9 specific laboratory markers were collected for preoperative measurement and postoperative measurements in the follow-up period. Preoperative measurement was defined as the value closest to the time of surgery within 1 week before surgery, and postoperative measurements were repeatedly measured within 12 months after surgery, with different intervals and times for individuals. The collected postoperative measurements excluded periods during which the patient was hospitalized after surgery or undergoing treatment. The collection of measurements for these 9 specific laboratory markers is labor-intensive, and ensuring the accuracy of these measurements can challenging. All participating facilities used standardized protocols to collect these measures. Comprehensive training was provided to all personnel involved in the data collection process to enhance their understanding of best practices. We also used a robust data management system to record and verify all results, along with regular audits to identify and address any discrepancies. By implementing these steps, we aimed to maintain high standards of accuracy and reliability in our data collection efforts.

**Figure 1.**
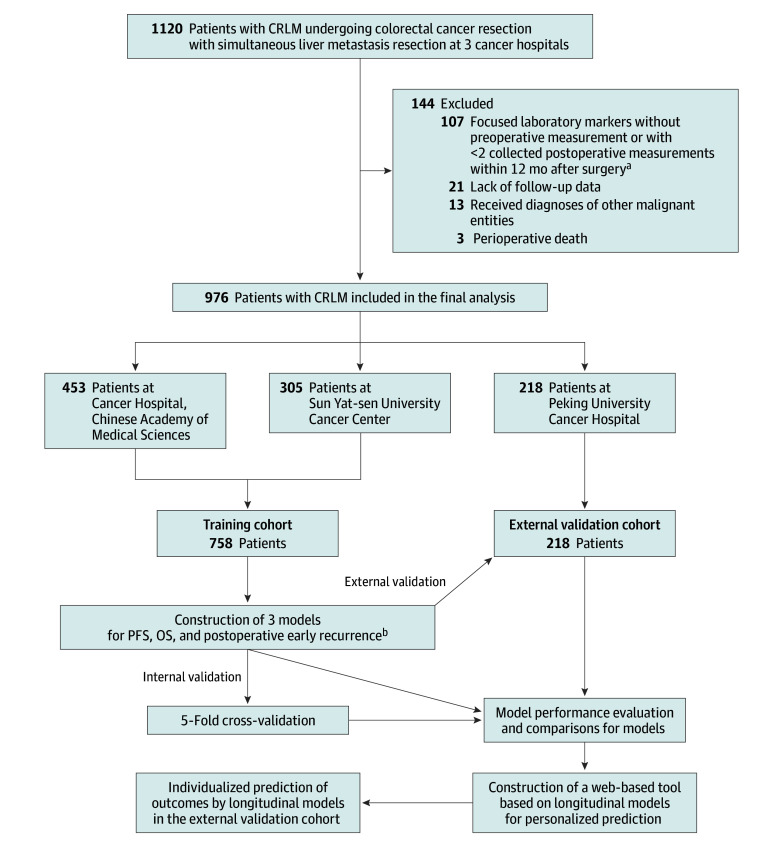
Flowchart for the Selection of Patients With Colorectal Cancer With Liver Metastases (CRLM) Receiving Simultaneous Resection and Overview of the Training and Validation Procedure for the Longitudinal Models ^a^Collected postoperative measurements exclude the period during which the patient was hospitalized after surgery or was undergoing treatment. ^b^Model A includes clinicopathologic characteristics. Model B includes clinicopathologic characteristics and 9 preoperative laboratory markers. Model C includes clinicopathologic characteristics and 9 longitudinal laboratory markers.

The flowchart patient enrollment is shown in Figure 1. Inclusion criteria were histologically confirmed liver metastases of colorectal adenocarcinoma and concurrent resection of CRC and liver metastases. Exclusion criteria included focused laboratory markers without preoperative measurement or with fewer than than 2 collected postoperative measurements within 12 months after surgery, perioperative mortality, presence of other malignant neoplasms, and absence of follow-up. Finally, a total of 758 patients from 2 independent cancer hospitals were included as the training cohort. Additionally, 218 patients from a third independent cancer hospital were designated as the external validation cohort.

### Follow-Up and Outcomes

After surgery, all patients had regular follow-up visits and were monitored for progression or recurrence in a standardized surveillance protocol incorporating multimodal imaging and tumor biomarker monitoring. Postoperative follow-up included contrast-enhanced computed tomography scans of the chest, abdomen, and pelvis; hepatic magnetic resonance imaging; and serum CEA level assessments. The initial postoperative evaluation was performed at 1 month after surgery, with subsequent comprehensive assessments conducted at 3-month intervals through our multidisciplinary oncology clinics. Outcomes included PFS, OS, and postoperative early recurrence. PFS was defined as the time from the date of simultaneous colorectal and liver resection to first documented disease progression or death due to any cause, whichever occurred first. Patients without PFS event were censored at their last follow-up assessment. OS was defined as the time from the date of simultaneous resection to death due to any cause. Patients without death were censored at their last known alive date. The cutoff value for defining postoperative early recurrence of CRLM was 13 months.^15^ Patients experiencing recurrence within 13 months were considered as experiencing postoperative early recurrence.

### Statistical Analysis

We adopted a functional survival forests framework that is capable of modeling multiple longitudinal data and survival information.^21^ The observed longitudinal values of a specific laboratory marker were assumed to come from a latent longitudinal process. As the longitudinal markers of interest were all associated with disease progression or death and are likely to be highly correlated, we applied the multivariate functional principal component (MFPC) approach^19^ to extract the overall trend and changing patterns of the markers while accounting for the correlations among them. The resulting MFPC scores, which could be considered as the weight of each extracted feature, were then used as predictors in the RSF model. This model was used for estimating patient risk of disease progression or death and allows for model reassessment as new longitudinal measurements become available. We investigated whether multivariate longitudinal laboratory markers, compared with clinicopathologic characteristics and baseline values of these laboratory markers, could enhance the predictive performance of disease progression or death. We did not thoroughly explore every possible combination of the markers, but focused on 3 variants: model A included clinicopathologic characteristics in the RSF model, model B included clinicopathologic characteristics and 9 preoperative laboratory markers, and model C included clinicopathologic characteristics and 9 longitudinal laboratory markers. For each model, a repeated 5-fold cross-validation^23^ (repeated 10 times) strategy was applied to the training dataset to ensure robust model performance estimation. The number of MFPC components was determined within each training iteration of the repeated 5-fold cross-validation procedure, using the criterion that selected components must collectively explain at least 97% of the total variance in the longitudinal laboratory markers.

The prognostic models were assessed in terms of discrimination and calibration using a suite of performance metrics, including area under the receiver operating characteristic curve (AUC) as discrimination measure^24^ and Brier score (BS).^25^ Error rate–based variable importance scores from the RSF models provided insights into importance and selection of clinical variables. We further showcased the models’ individualized dynamic prognostic capabilities via predicting PFS and OS for specific patients randomly selected from the external validation dataset. Dynamic prognosis has the advantage of including the most current information of markers in estimating the updated MFPC scores and subsequently update the risk prediction. *P* values were 2-sided, and statistical significance was set at *P* < .05. Data were analyzed using R version 4.1.1 (R Project for Statistical Computing) from June 2024 to June 2025.

## Results

### Patient Characteristics and Outcomes

A total of 976 patients (median [IQR] age, 59 [51-65] years; 612 [62.7%] male) were eligible for this study, including 758 patients in the training cohort (median [IQR] age, 59 [52-66] years; 487 [64.2%] male) and 218 patients in the validation cohort (median [IQR] age, 58 [49-64] years; 125 [57.3%] male). The characteristics of patients are outlined in Table 1. In the training cohort, 31 692 measurements of 11 hematological markers were collected; ultimately, 24 992 measurements of 9 specific laboratory markers were included in the longitudinal model. The training cohort had a median (IQR) follow-up of 42.6 (40.2-45.1) months. During follow-up, a total of 465 patients (61.3%) experienced recurrence, with a median (IQR) PFS of 17.0 (14.4-15.6) months, 316 patients (41.6%) experienced postoperative early recurrence, and 225 patients (29.7%) died. Among 218 patients in the external validation cohort, 9203 measurements of 11 hematological markers and 7198 measurements of 9 specific laboratory markers were included. The external validation cohort had a median (IQR) follow-up of 35.9 (32.2-39.5) months. During follow-up, a total of 151 patients (69.2%) experienced recurrence, with a median PFS of 10.0 (8.5-11.5) months, 132 patients (60.6%) experienced postoperative early recurrence, and 89 patients (40.8%) died, with a median (IQR) OS of 41.0 (33.1-48.9) months. The Kaplan-Meier curves for PFS and OS for the training and validation cohorts are shown in eFigure 1 in Supplement 1.

**Table 1.** Clinicopathologic Characteristics of Patients With Colorectal Cancer With Liver Metastases Receiving Simultaneous Resection

Variables	Patients, No. (%)		
	Total (N = 976)	Cohort	
		Training (n = 758)	External validation (n = 218)
Age, median (IQR), y	59 (51-65)	59 (52-66)	58 (49-64)
Sex			
Female	364 (37.3)	271 (35.8)	93 (42.7)
Male	612 (62.7)	487 (64.2)	125 (57.3)
Any comorbidity	409 (41.9)	301 (39.7)	108 (49.5)
ASA score 1-2	905 (92.7)	690 (91.0)	215 (98.6)
Preoperative laboratory measures, median (IQR)			
CEA, ng/mL	8.39 (3.73-25.71)	8.80 (3.81-26.9)	7.01 (3.61-22.7)
CA19-9, U/mL	22.5 (10.7-55.6)	21.9 (10.2-56.6)	22.8 (12.5-52.1)
APRI	0.264 (0.175-0.407)	0.244 (0.163-0.370)	0.340 (0.235-0.541)
S-index	0.087 (0.050-0.149)	0.083 (0.047-0.145)	0.102 (0.060-0.156)
FIB-4	1.40 (0.957-2.12)	1.26 (0.882-1.96)	1.82 (1.24-2.46)
GGT, U/L	33.0 (20.7-49.0)	32.0 (20.0-48.0)	34.0 (22.0-51.8)
NLR	2.12 (1.53-2.94)	2.10 (1.55-2.92)	2.24 (1.48-3.21)
RDW-SD, fL	47.7 (42.9-54.2)	46.5 (42.4-53.4)	50.0 (45.5-56.7)
RDW-CV, %	14.4 (13.0-16.4)	14.1 (12.8-16.1)	15.2 (13.8-16.9)
Preoperative chemotherapy, No. (%)	621 (63.6)	434 (57.3)	187 (85.8)
Primary site in colon	669 (68.5)	482 (63.6)	187 (85.8)
Right hemicolon	269 (27.6)	188 (24.8)	81 (37.2)
Bilobar liver distribution	429 (44.0)	303 (40.0)	126 (57.8)
Liver metastases, median (IQR), No.	2 (1-4)	2 (1-3)	3 (2-6)
Diameter of liver metastases, median (IQR), cm	2.3 (1.5-3.5)	2.3 (1.5-3.5)	2.4 (1.5-3.5)
Extrahepatic metastases	110 (11.3)	66 (8.7)	44 (20.2)
Major liver resection	334 (34.2)	296 (39.1)	38 (17.4)
R0 resection	762 (78.1)	595 (78.5)	167 (76.6)
Poor differentiation	240 (24.6)	209 (27.6)	31 (14.2)
T3-T4 stage	886 (90.8)	686 (90.5)	200 (91.7)
N1-N2 stage	637 (65.3)	484 (63.9)	153 (70.2)
*KRAS* mutation	302 (30.9)	216 (28.5)	86 (39.4)
Adjuvant chemotherapy	688 (70.5)	509 (67.2)	179 (82.1)

Abbreviations: APRI, aspartate aminotransferase to platelet ratio index; CA, carbohydrate antigen; CEA, carcinoembryonic antigen; CV, coefficient of variation; FIB-4, Fibrous-4 index; GGT, γ-glutamyl transferase; NLR, neutrophil-to-lymphocyte ratio; RDW, red blood cell distribution width.

SI conversion factors: To convert CEA to micrograms per liter, multiply by 1; GGT to microkatals per liter, multiply by 0.0167.

### Feature Extraction of Multiple Longitudinal Markers

The curves of 9 longitudinal laboratory markers of patients with and without recurrence and patients who were alive or had died by the final follow-up are shown in eFigure 2 in Supplement 1. Patients who experienced recurrence tended to have higher postoperative CA19-9, CEA, FIB-4, APRI, RDW-CV, RDW-SD, and NLR within 12 months after surgery. Patients who died tended to have higher postoperative CEA, GGT, APRI, and S-index within 12 months after surgery. eFigure 2 in Supplement 1 provides a descriptive overview of marker trends but does not adjust for other covariates; therefore, observed differences between groups should be interpreted as crude comparisons only. We selected the first 6 MFPC components, which accounted for 99% total variation in the 9 longitudinal markers. eFigure 3 in Supplement 1 displays the first 6 MFPCs for the 9 longitudinal markers for PFS and OS. The relative importance of the variables in the longitudinal model for prediction of PFS and for OS is shown in eFigure 4 in Supplement 1. The principal components for the longitudinal 9 laboratory markers were important predictors for PFS and OS.

### Performance of Prognostic Models for PFS and Early Recurrence

The performance measures for the prognostic models for PFS and early recurrence are shown in Table 2. For cross-validation, AUCs for model A were 0.692 at 1 year, 0.700 at 3 years, and 0.704 at 5 years. The BSs for model A were 0.216 at 1 year, 0.204 at 3 years, and 0.193 at 5 years. The performance of model B was enhanced by including the 9 preoperative laboratory markers. Furthermore, the performance of model C was improved by including the 9 longitudinal laboratory markers. The AUCs for model C were 0.737 at 1 year, 0.737 at 3 years, and 0.726 at 5 years. The BSs for the model C were 0.207 at 1 year, 0.198 at 3 years, and 0.193 at 5 years. For prediction of early recurrence, the AUC of model C was better than models A and B (0.740 vs 0.697 vs 0.701), and the BS of model C was lower than those for models A and B (0.209 vs 0.218 vs 0.218).

**Table 2.** AUCs and Brier Scores of 3 Model for the Prediction of 1-, 3-, 5-Year PFS and Early Recurrence in Cross-Validation and External Validation Cohort

Model	PFS AUC			PFS Brier score			Early recurrence	
	1-y	3-y	5-y	1-y	3-y	5-y	AUC	Brier score
**Cross-validation, estimate**								
A^a^	0.692	0.700	0.704	0.216	0.204	0.193	0.697	0.218
B^b^	0.696	0.710	0.724	0.216	0.204	0.193	0.701	0.218
C^c^	0.737	0.737	0.726	0.207	0.198	0.193	0.740	0.209
**External validation cohort, estimate (95% CI)**								
A^a^	0.712 (0.641-0.774)	0.763 (0.684-0.829)	0.726 (0.634-0.811)	0.249 (0.236-0.262)	0.206 (0.195-0.221)	0.142 (0.133-0.156)	0.705 (0.637-0.769)	0.247 (0.240-0.266)
B^b^	0.740 (0.675-0.798)	0.769 (0.684-0.843)	0.759 (0.674-0.842)	0.246 (0.235-0.258)	0.202 (0.193-0.217)	0.139 (0.131-0.153)	0.729 (0.663-0.790)	0.243 (0.238-0.262)
C^c^	0.796 (0.740-0.848)	0.837 (0.768-0.899)	0.850 (0.780-0.914)	0.246 (0.236-0.261)	0.205 (0.193-0.218)	0.142 (0.132-0.153)	0.791 (0.731-0.848)	0.243 (0.239-0.265)

Abbreviations: AUC, area under the receiver operating characteristic curve; PFS, progression-free survival.

^a^ Includes clinicopathologic characteristics.

^b^ Includes clinicopathologic characteristics and 9 preoperative laboratory markers.

^c^ Includes clinicopathologic characteristics and 9 longitudinal laboratory markers.

In the external validation cohort, AUCs for model C were 0.796 (95% CI, 0.740-0.848) at 1 year, 0.837 (95% CI, 0.768-0.899) at 3 years, and 0.850 (95% CI, 0.780-0.914) at 5 years. The BSs for model C were 0.246 (95% CI, 0.236-0.261) at 1 year, 0.205 (95% CI, 0.193-0.218) at 3 years, and 0.142 (95% CI, 0.132-0.153) at 5 years. The AUC for postoperative early recurrence in model C was 0.791 (95% CI, 0.731-0.848), and the BS was 0.243 (95% CI, 0.239-0.265). Furthermore, calibration plots of model C for PFS prediction demonstrated good calibration between the predictions and observations (eFigure 5 in Supplement 1). Model C showed good performance for predicting PFS in the external validation cohort.

### Performance of Prediction Models for OS

The performance of the OS prediction models is summarized in Table 3. During cross-validation, the AUCs for model A were 0.713 at 1 year, 0.679 at 3 years, and 0.669 at 5 years. The corresponding BSs for model A were 0.038 at 1 year, 0.184 at 3 years, and 0.225 at 5 years. The predictive performance improved in model B with the addition of 9 preoperative laboratory markers. Furthermore, the prediction accuracy was enhanced in model C by incorporating 9 longitudinal laboratory markers. The AUCs for Model C were 0.786 at 1 year, 0.748 at 3 years, and 0.743 at 5 years, with BSs of 0.036 at 1 year, 0.172 at 3 years, and 0.205 at 5 years.

**Table 3.** AUCs and Brier Scores of 3 Model for the Prediction of 1-, 3-, 5-Year Overall Survival in Cross-Validation and External Validation Cohort

Models	AUC			Brier scores		
	1-y	3-y	5-y	1-y	3-y	5-y
**Cross-validation, estimate**						
A^a^	0.713	0.679	0.669	0.038	0.184	0.225
B^b^	0.705	0.689	0.688	0.038	0.183	0.220
C^c^	0.786	0.748	0.743	0.036	0.172	0.205
**External validation cohort, estimate (95% CI)**						
A^a^	0.717 (0.586-0.833)	0.599 (0.514-0.684)	0.693 (0.589-0.794)	0.046 (0.045-0.048)	0.170 (0.167-0.193)	0.137 (0.132-0.162)
B^b^	0.727 (0.601-0.835)	0.597 (0.510-0.681)	0.683 (0.578-0.788)	0.047 (0.045-0.048)	0.171 (0.167-0.190)	0.136 (0.130-0.157)
C^c^	0.849 (0.768-0.914)	0.741 (0.667-0.815)	0.753 (0.656-0.849)	0.047 (0.045-0.048)	0.178 (0.168-0.195)	0.144 (0.133-0.165)

Abbreviation: AUC, area under the receiver operating characteristic curve.

^a^ Includes clinicopathologic characteristics.

^b^ Includes clinicopathologic characteristics and 9 preoperative laboratory markers.

^c^ Includes clinicopathologic characteristics and 9 longitudinal laboratory markers.

In the external validation cohort, model C demonstrated AUCs of 0.849 (95% CI, 0.768-0.914) at 1 year, 0.741 (95% CI, 0.667-0.815) at 3 years, and 0.753 (95% CI, 0.656-0.849) at 5 years. The BSs for model C were 0.047 (95% CI, 0.045-0.048) at 1 year, 0.178 (95% CI, 0.168-0.195) at 3 years, and 0.144 (95% CI, 0.133-0.165) at 5 years. Overall, model C outperformed both models A and B in predictive performance. In addition, the calibration plots for model C demonstrated good agreement between the model’s predictions and actual outcomes (eFigure 5 in Supplement 1).

### Personalized Dynamic Prediction

**Figure 2.**
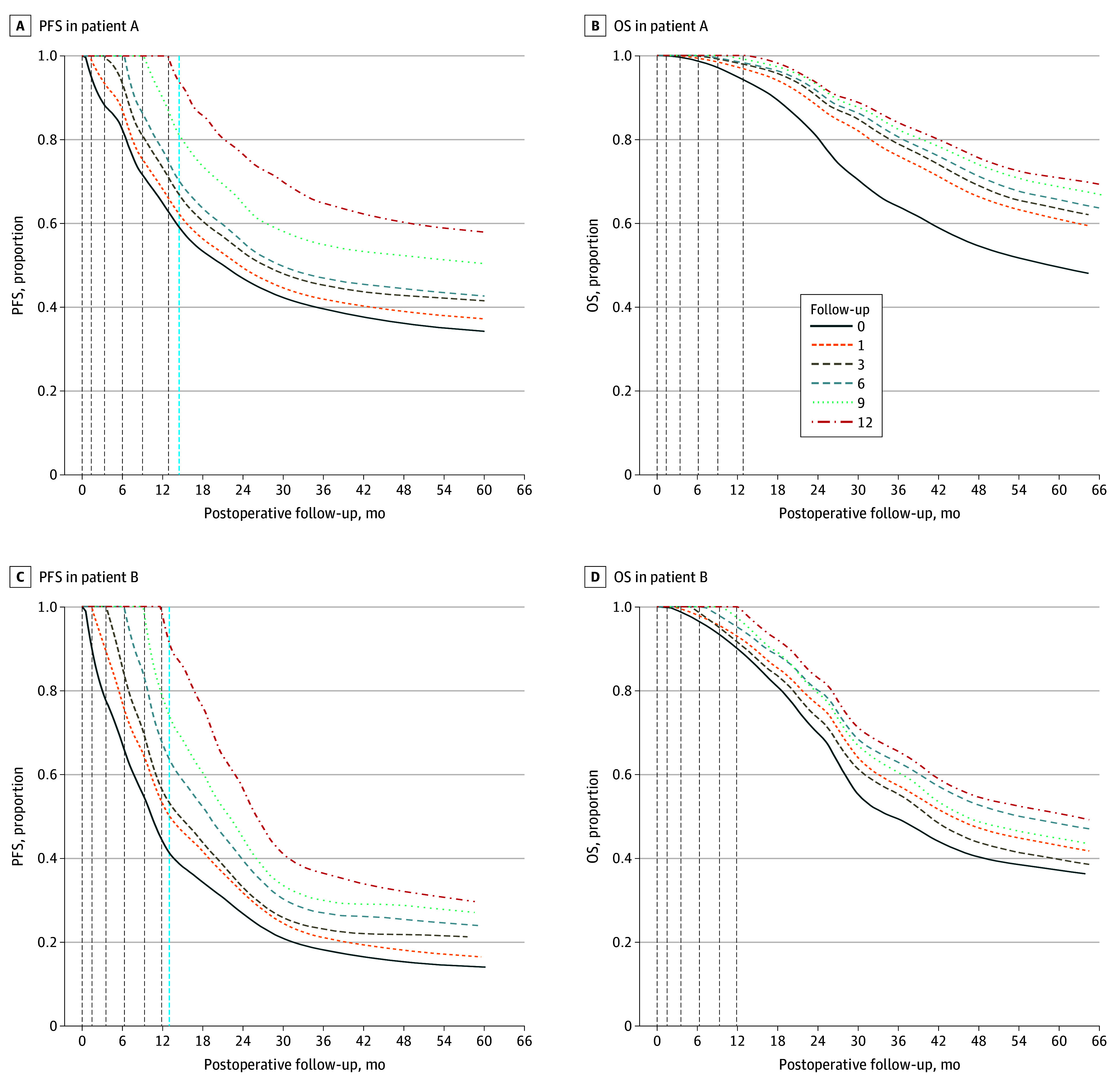
Individualize Dynamic Prediction of Progression-Free Survival (PFS) and Overall Survival (OS) Risk Profile in Patient A and Patient B Each curve represents the model’s prediction based on data entered at each follow-up assessment. Dashed vertical lines indicate follow-up visits when data were collected.

An advantage of the functional survival forest framework that we used is its ability to update personalized prognosis as new longitudinal data become available. We examined how personalized dynamic predictions for PFS and OS could be made for 2 individuals (patients A and B) randomly selected from the external validation cohort (Figure 2). Patient A had nonbilobar liver distribution, single liver metastases, and diameter of liver metastases less than 3 cm. Patient B had bilobar liver distribution, multiple liver metastases, and diameter of liver metastases greater than 3 cm. Each patient was examined 6 times in the time window of 0 to 12 months. The patient’s survival prognosis was updated at each visit via incorporation new laboratory measurements collected at that visit. Predicted PFS and OS estimates were made until approximately 60 months, with survival curves plotted from the cubic spline–smoothed trajectories (Figure 2). Note that the estimate indicates a conditional probability of being event-free at a future time point, given that the patient was event-free at their current visit. As the landmark time extends, more follow-up laboratory measurements are incorporated in the risk prediction model, leading to updated survival probabilities. For patient A, the predicted probabilities of remaining event-free (ie, no disease progression or death) increased notably after baseline visits, particularly after the 12-month visit. This suggests that, based on the additional laboratory data collected at postbaseline visits, patient A was likely to be in a relatively stable condition, with reduced risk of disease progression, compared with the initial risk prediction made at baseline. In contrast, while patient B also exhibited a lower predicted risk for future recurrence or death with incorporation of new information collected at follow-up visits, the magnitude of this risk reduction was not as pronounced as that of patient A (Figure 2). These findings suggest that patient B was at a higher risk of progression and mortality and therefore should be monitored closely. eTable 1 and eTable 2 in Supplement 1 suggest consistent model performances (in the external validation dataset) for the future risk predictions that updated at different time points (eg, 3, 6, 9, and 12 months), given the patients were event-free up to the predicted time point.

To demonstrate the potential use of the models for personalized dynamic predictions in the clinical setting, we developed a web-based calculator.^22^ A screenshot of the user interface is presented in eFigure 6 in Supplement 1. The calculator requires the patient’s baseline characteristics and 9 longitudinal laboratory markers (missing values are allowed) until the current visit. The calculator then estimates the future risk profile of the patient, eg, risk of PFS and OS.

## Discussion

This prognostic study constructed dynamic models innovatively incorporating 9 specific longitudinal laboratory markers to estimate prognosis among patients with CRLM undergoing simultaneous resection. Furthermore, a web-based tool was developed to facilitate the practical application of the dynamic prognostic models for new patients in a clinical setting.

Elevated preoperative levels of CEA and CA19-9 in patients with CRC are associated with worse survival,^10,11,26^ establishing these markers as crucial for survival prediction in CRC models. Previous studies have demonstrated that incorporating tumor markers improves the accuracy of preoperative prognostic models.^27,28^ In addition, among patients with stages I to III CRC, models additionally integrating perioperative longitudinal CEA and CA19-9 measurements exhibit markedly enhanced prognostic accuracy.^29^ However, the significance of dynamic change of longitudinal preoperative and postoperative CEA and CA19-9 for the estimation of prognoses among patients with CRLM is unclear. To our knowledge, this is the first study to examine whether a preoperative model incorporating perioperative dynamic changes in CEA and CA19-9 substantially improves predictive capability for CRLM, thereby reinforcing the rationale for continued postoperative monitoring of tumor markers. Furthermore, preoperative nontumor hematological markers and their composite indices, such as NLR, reflecting the balance between protumor and antitumor inflammation interactions^18^; APRI, S-index, FIB-4, and GGT, assessing the tumor fibrotic microenvironment^12,15,16,17^; and RDW-SD and RDW-CV, evaluating the inflammatory response in tumors,^13,14^ were found to be associated with prognosis in patients with CRLM receiving resection. While the roles of these markers and indices in preoperative models are well-recognized, the association of their changes in postoperative follow-ups with prognoses has not been thoroughly explored. In this study, we advanced the models by additionally incorporating longitudinal tumor markers and nontumor indicators, resulting in more comprehensive longitudinal prediction models and demonstrating superior predictive capability. In addition, in an external validation cohort, the longitudinal prediction models showed comparable predictive performance as in the training cohort, which demonstrates the general applicability of the model. The 1-year, 3-year, and 5-year AUCs of the longitudinal survival models developed in this study outperformed those of other models, including random forest models and modified clinical scores reported in previous research.^6,7,8,9^ An additional benefit of dynamic prediction model is its broad applicability and cost-effectiveness. The model uses longitudinal laboratory markers, which are standard metrics in the postoperative monitoring of patients with CRLM. Given that these markers are routinely available in most hospitals, they are affordable and do not incur extra costs for patients. This accessibility ensures that a larger number of patients can take advantage of the prognostic power of this model. In addition, we have developed a user-friendly web-based prediction tool^22^ that integrates patients’ baseline characteristics and longitudinal laboratory markers for practical clinical use. The tool features an intuitive interface allowing clinicians to input individual patient data and promptly obtain dynamic risk predictions that update with each new laboratory measurement. This real-time functionality supports clinicians in making informed decisions by providing precise, personalized risk assessments. For example, in routine clinical scenarios, a clinician could use the tool to monitor changes in a patient’s risk profile during treatment, enabling timely adjustments to therapy or follow-up schedules. Such practical applications highlight the tool’s potential to enhance patient treatment through tailored interventions guided by continuously updated risk predictions.

For the dynamic prediction of patient’s risk of recurrence or death, we adopt a functional survival forests framework that is capable of incorporating longitudinal information. MFPC methods^19^ were used to extract informative features from the trajectories of longitudinal outcomes. These methods are able to deal with both temporal correlation within patients’ repeated measures and associations between different longitudinal outcomes. The resulting MFPC scores were then included as covariates in the RSF. The advantages of RSF are that it is nonparametric, which requires no assumptions about the underlying distribution, and it is able to identify interactions between variables without having to explicitly model them. This flexibility allows RSF to have more robust discrimination and calibration metrics in dynamic predictions. The approach was previously evaluated in a study by Li et al^20^ the Alzheimer disease setting. The findings of Li et al^20^ suggest that the functional survival forest model was able to better identify patterns in the data, leading to higher predictive power for the progression of disease and mortality.^20^

### Limitations

Our study has several limitations. First, the retrospective design may introduce sample selection bias. Although our prediction models demonstrated consistent performance in an independent validation cohort using a moderately sized dataset, our conclusions could be bolstered by assessing these models in a larger cohort or, ideally, through well-designed prospective, controlled trials. Second, the computed MFPC scores and component functions may lack clinical interpretability, and not all scores are significantly associated with the likelihood of disease progression and death. While we assessed variable importance and variable selection for the MFPC scores, the specific contributions of individual laboratory markers to each principal component remain ambiguous. Future studies could explore adaptive or outcome-guided component selection strategies to enhance both model performance and interpretability. It is also possible that further optimization of model hyperparameters could yield incremental improvements in predictive accuracy. Third, although our prediction models incorporated a comprehensive set of clinicopathologic and laboratory variables, we recognize that unmeasured or residual confounding may persist, as not all possible prognostic factors could be included. This limitation is inherent to retrospective observational studies and may affect the model’s predictive accuracy in certain subgroups. Future research should aim to incorporate additional relevant variables and to validate the model in broader cohorts to further enhance its generalizability and reliability. Despite these limitations, our findings suggest that this is a promising avenue for developing more accurate predictive models in the future.

## Conclusions

This retrospective prognostic study developed dynamic models incorporating multiple longitudinal laboratory markers and created a web-based tool for practical application in clinical settings for patients with CRLM receiving simultaneous resection. The tool demonstrated promising performance, offering potential for precise and individualized decision-making in CRLM care.

## Notes

Supplement 1. **eFigure 1.** Progression-free survival and overall survival Kaplan-Meier curve for the training cohort and validation cohort **eFigure 2.** LOESS-smoothed trajectories of 9 specific laboratory markers over time for of no recurrence and recurrence patients and of alive and death patients **eFigure 3.** The first 6 eigenfunctions for 9 specific laboratory markers based on MFPCA of the longitudinal model for the prediction of PFS and OS **eFigure 4.** Variable importance of longitudinal models for the prediction of PFS and OS **eFigure 5.** Calibration plots of Model C for predicting 1-year, 3-year, and 5-year PFS and OS and for the average predicted probability of PFS and OS against observed risk of PFS and OS over time **eFigure 6.** Screenshot of the user interface for Dynamic Prediction of Health Outcome of Patients with Colorectal Liver Metastases Receiving Simultaneous Resection **eTable 1.** Dynamic performance for the prediction of 1-, 3-, 5-year PFS and OS (from baseline) at different prediction time points in external validation cohort **eTable 2.** Dynamic performance for the prediction of PFS and OS (from prediction time points) in external validation cohort

Supplement 2.
Data Sharing Statement
