# Supplementary material for: Dynamic Prognostic Models for Colorectal Cancer With Liver Metastases
Source: JAMA Netw Open. 2025 Aug 27;8(8):e2529093. doi: 10.1001/jamanetworkopen.2025.29093 (PMC12391993; doi:10.1001/jamanetworkopen.2025.29093)
Supplement: Supplement 1. — eFigure 1. Progression-free survival and overall survival Kaplan-Meier curve for the training cohort and validation cohort eFigure 2. LOESS-smoothed trajectories of 9 specific laboratory markers over time for of no recurrence and recurrence patients and of alive and death patients eFigure 3. The first 6 eigenfunctions for 9 specific laboratory markers based on MFPCA of the longitudinal model for the prediction of PFS and OS eFigure 4. Variable importance of longitudinal models for the prediction of PFS and OS eFigure 5. Calibration plots of Model C for predicting 1-year, 3-year, and 5-year PFS and OS and for the average predicted probability of PFS and OS against observed risk of PFS and OS over time eFigure 6. Screenshot of the user interface for Dynamic Prediction of Health Outcome of Patients with Colorectal Liver Metastases Receiving Simultaneous Resection eTable 1. Dynamic performance for the prediction of 1-, 3-, 5-year PFS and OS (from baseline) at different prediction time points in external validation cohort eTable 2. Dynamic performance for the prediction of PFS and OS (from prediction time points) in external validation cohort [file jamanetwopen-e2529093-s001.pdf]

Supplemental Online Content

Chen Q, Deng Y, Wang K, et al. Dynamic prognostic models for colorectal cancer with liver metastases. *JAMA Netw Open*. 2025;8(8):e2529093.
doi:10.1001/jamanetworkopen.2025.29093

eFigure 1. Progression-free survival and overall survival Kaplan-Meier curve for the training cohort and validation cohort
eFigure 2. LOESS-smoothed trajectories of 9 specific laboratory markers over time for of no recurrence and recurrence patients and of alive and death patients
eFigure 3. The first 6 eigenfunctions for 9 specific laboratory markers based on MFPCA of the longitudinal model for the prediction of PFS and OS
eFigure 4. Variable importance of longitudinal models for the prediction of PFS and OS
eFigure 5. Calibration plots of Model C for predicting 1-year, 3-year, and 5-year PFS and OS and for the average predicted probability of PFS and OS against observed risk of PFS and OS over time
eFigure 6. Screenshot of the user interface for Dynamic Prediction of Health Outcome of Patients with Colorectal Liver Metastases Receiving Simultaneous Resection
eTable 1. Dynamic performance for the prediction of 1-, 3-, 5-year PFS and OS (from baseline) at different prediction time points in external validation cohort
eTable 2. Dynamic performance for the prediction of PFS and OS (from prediction time points) in external validation cohort

This supplemental material has been provided by the authors to give readers additional information about their work.

© 2025 Chen Q et al. *JAMA Network Open*.

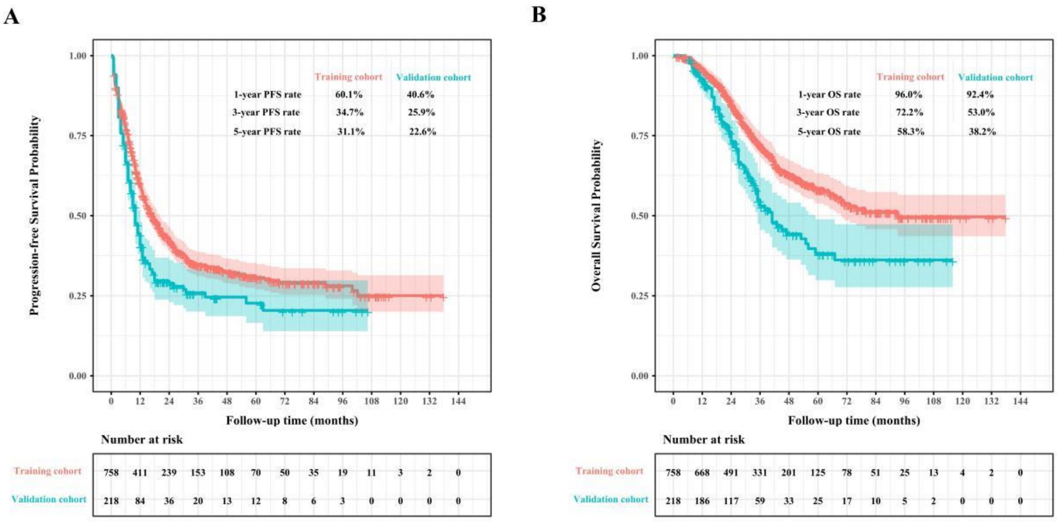

Supplementary Figure 1. Progression-free survival **(A)** and overall survival **(B)** Kaplan-Meier curve for the training cohort and validation cohort.

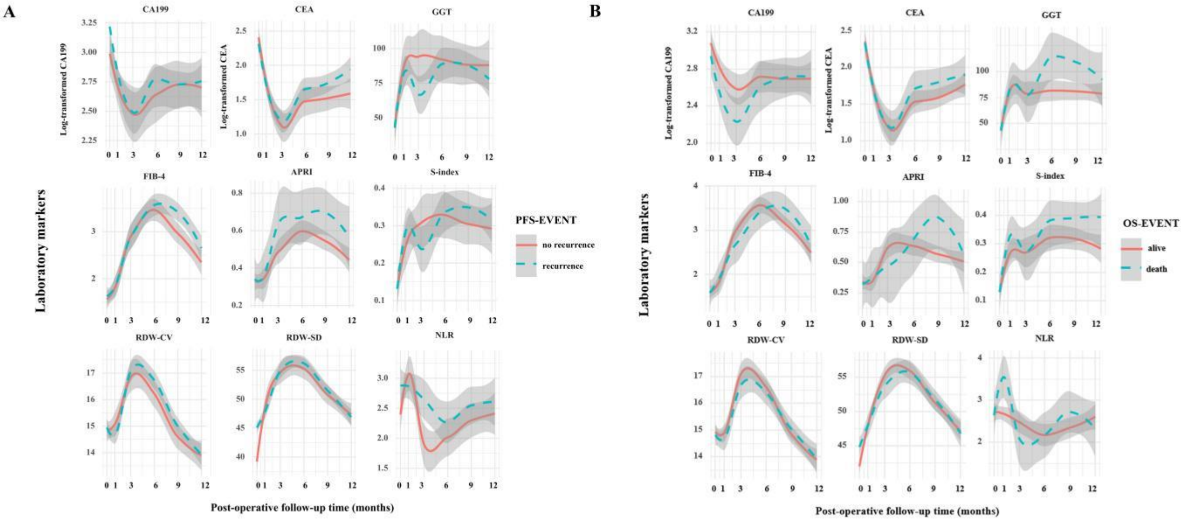

Supplementary Figure 2. LOESS-smoothed trajectories of 9 specific laboratory markers over time for **(A)** of no recurrence and recurrence patients and **(B)** of alive and death patients. The shaded regions are 95% pointwise confidence intervals.

© 2025 Chen Q et al. *JAMA Network Open*.

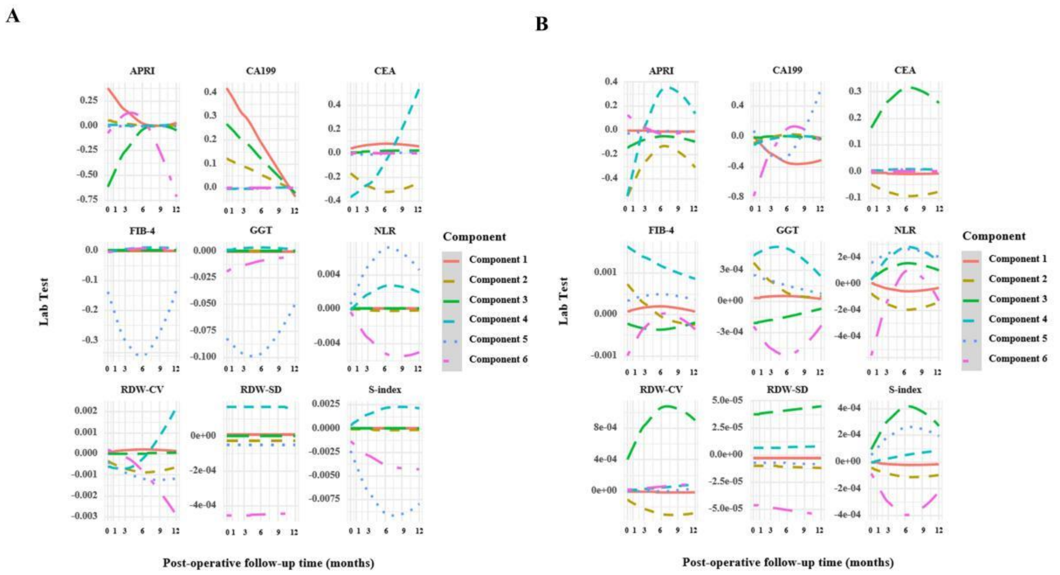

Supplementary Figure 3. The first six eigenfunctions for 9 specific laboratory markers based on MFPCA of the longitudinal model for the prediction of PFS (**A**) and OS (**B**)

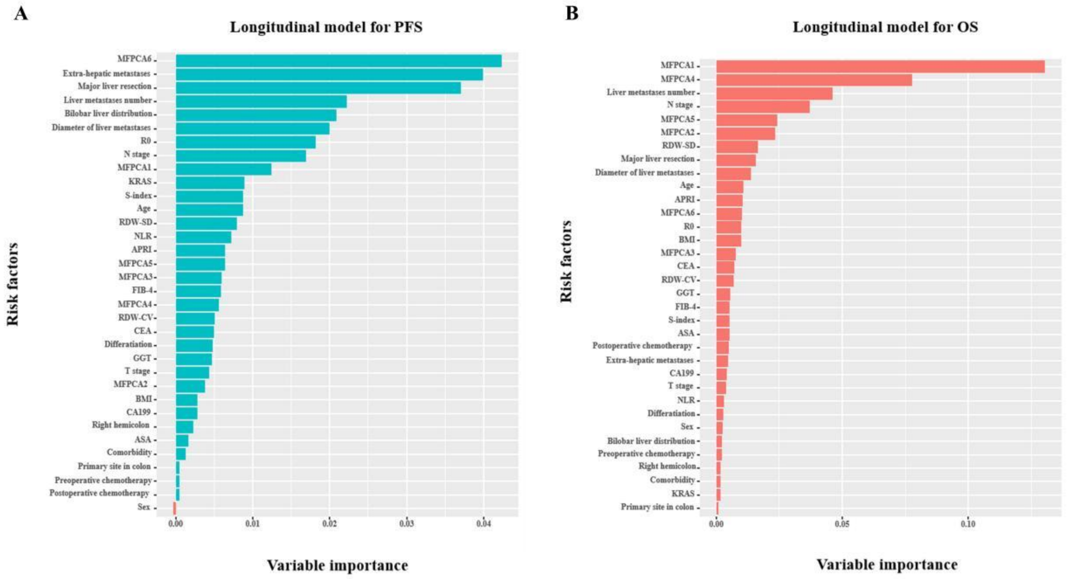

Supplementary Figure 4. Variable importance of longitudinal models for the prediction of PFS (**A**) and OS (**B**)

© 2025 Chen Q et al. *JAMA Network Open*.

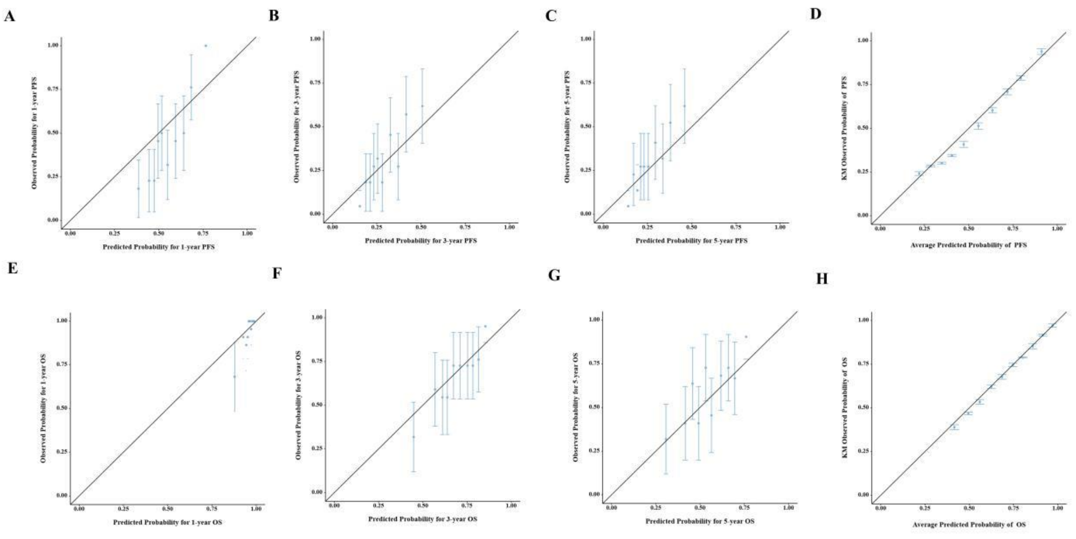

Supplementary Figure 5. In external validation cohort, calibration plots of Model C for predicting 1-year PFS (**A**), predicting 3-year PFS (**B**), predicting 5-year PFS (**C**) and for the average predicted probability of PFS against observed risk of PFS over time (**D**); Calibration plots of Model C for predicting 1-year OS (**E**), predicting 3-year OS (**F**), predicting 5-year OS (**G**) and the average predicted probability of OS against observed risk of OS over time (**H**).

© 2025 Chen Q et al. *JAMA Network Open*.

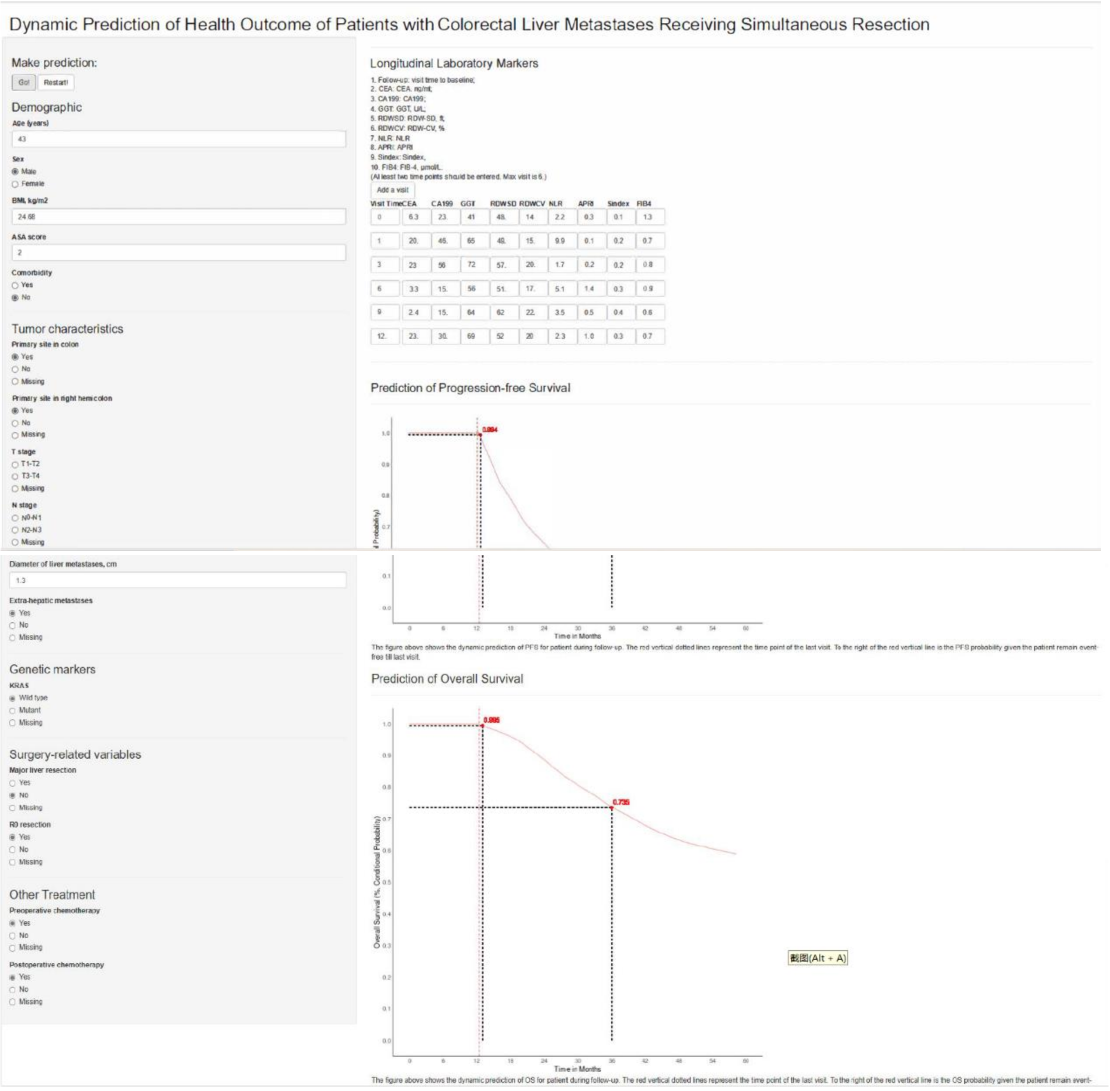

Supplementary Figure 6. Screenshot of the user interface for "Dynamic Prediction of Health Outcome of Patients with Colorectal Liver Metastases Receiving Simultaneous Resection".

© 2025 Chen Q et al. *JAMA Network Open*.

Supplementary Table 1. Dynamic performance for the prediction of 1-, 3-, 5-year PFS and OS (from baseline) at different prediction time points in external validation cohort

Models	Prediction Time Point (months)	AUC (95%CI)			Brier scores (95%CI)		
		1-year PFS	3-year PFS	5-year PFS	1-year PFS	3-year PFS	5-year PFS
**Model B**	**0**	0.740 (0.675-0.798)	0.769 (0.684-0.843)	0.759 (0.674-0.842)	0.246 (0.235-0.258)	0.202 (0.193-0.217)	0.139 (0.131-0.153)
**Model C**	**3**	0.785 (0.727-0.842)	0.822 (0.747-0.885)	0.833 (0.751-0.907)	0.248 (0.234-0.261)	0.234 (0.208-0.236)	0.166 (0.142-0.168)
	**6**	0.780 (0.720-0.842)	0.797 (0.708-0.872)	0.809 (0.717-0.889)	0.241 (0.224-0.253)	0.242 (0.228-0.263)	0.175 (0.152-0.183)
	**9**	0.732 (0.634-0.817)	0.744 (0.641-0.840)	0.757 (0.653-0.863)	0.237 (0.201-0.234)	0.272 (0.220-0.261)	0.225 (0.179-0.22)
	**12**	not applicable	0.674 (0.542-0.798)	0.687 (0.545-0.822)	0.187 (0.182-0.220)	0.184 (0.170-0.214)	0.097 (0.083-0.112)
**Models**	**Prediction Time Point (months)**	**1-year OS**	**3-year OS**	**5-year OS**	**1-year OS**	**3-year OS**	**5-year OS**
**Model B**	**0**	0.727 (0.601-0.835)	0.597 (0.510-0.681)	0.683 (0.578-0.788)	0.047 (0.045-0.048)	0.171 (0.167-0.190)	0.136 (0.130-0.157)
**Model C**	**3**	0.850 (0.768-0.915)	0.742 (0.667-0.815)	0.753 (0.656-0.849)	0.047 (0.045-0.048)	0.178 (0.168-0.195)	0.144 (0.133-0.165)
	**6**	0.845 (0.757-0.916)	0.737 (0.658-0.812)	0.750 (0.653-0.840)	0.046 (0.046-0.049)	0.179 (0.170- 0.197)	0.148 (0.136-0.168)
	**9**	0.926 (0.871-0.971)	0.726 (0.647-0.799)	0.742 (0.642-0.835)	0.052 (0.049-0.053)	0.206 (0.181-0.211)	0.166 (0.146-0.181)
	**12**	not applicable	0.706 (0.627-0.784)	0.730 (0.623-0.825)	0.049 (0.048-0.051)	0.192 (0.187-0.217)	0.168 (0.152-0.188)

Model B: model incorporating with clinicopathologic characteristics and preoperative 9 laboratory markers; Model C: model incorporating with clinicopathologic characteristics and longitudinal 9 laboratory markers

© 2025 Chen Q et al. *JAMA Network Open*.

Supplementary Table 2. Dynamic performance for the prediction of PFS and OS (from prediction time points) in external validation cohort

Models	Prediction Time Point (months)	AUC (95%CI)		Brier scores (95%CI)	
		12-month ahead PFS	24-month ahead PFS	12-month ahead PFS	24-month ahead PFS
**Model C**	**3**	0.759 (0.695-0.819)	0.744 (0.662-0.822)	0.253 (0.236-0.264)	0.277 (0.242-0.272)
	**6**	0.74 (0.669-0.811)	0.716 (0.629-0.801)	0.242 (0.230-0.261)	0.254 (0.248-0.283)
	**9**	0.657 (0.566-0.748)	0.71 (0.605-0.813)	0.263 (0.216-0.255)	0.267 (0.219-0.258)
	**12**	0.614 (0.490-0.732)	0.674 (0.542-0.798)	0.212 (0.199-0.245)	0.184 (0.170-0.214)
**Models**	**Prediction Time Point (months)**	**12-month ahead OS**	**24-month ahead OS**	**12-month ahead OS**	**24-month ahead OS**
**Model C**	**3**	0.855 (0.786-0.915)	0.815 (0.751-0.871)	0.067 (0.063-0.068)	0.154 (0.138-0.157)
	**6**	0.805 (0.723-0.873)	0.756 (0.680-0.823)	0.076 (0.073-0.080)	0.169 (0.163-0.188)
	**9**	0.774 (0.698-0.854)	0.761 (0.687-0.828)	0.086 (0.077-0.088)	0.196 (0.176-0.203)
	**12**	0.763 (0.685-0.832)	0.706 (0.627-0.784)	0.111 (0.110-0.126)	0.192 (0.187-0.217)

Model C: model incorporating with clinicopathologic characteristics and longitudinal 9 laboratory markers

© 2025 Chen Q et al. *JAMA Network Open*.
