# Supplementary material for: Dynamic Prognostic Models for Colorectal Cancer With Liver Metastases
Source: JAMA Netw Open. 2025 Aug 27;8(8):e2529093. doi: 10.1001/jamanetworkopen.2025.29093 (PMC12391993; doi:10.1001/jamanetworkopen.2025.29093)
Supplement: Supplement 2. — Data Sharing Statement [file jamanetwopen-e2529093-s002.pdf]

Data Sharing Statement

Chen. Dynamic Prognostic Models for Colorectal Cancer With Liver Metastases. *JAMA Netw Open*. Published August 27, 2025. doi:10.1001/jamanetworkopen.2025.29093

Data
Data available: No

Additional Information
Explanation for why data not available: The data supporting the findings of this study are available upon request from the corresponding author. Due to privacy restrictions, these data are not publicly accessible.
